# Glutamate Levels and Resting Cerebral Blood Flow in Anterior Cingulate Cortex Are Associated at Rest and Immediately Following Infusion of S-Ketamine in Healthy Volunteers

**DOI:** 10.3389/fpsyt.2018.00022

**Published:** 2018-02-06

**Authors:** Kirsten Borup Bojesen, Kasper Aagaard Andersen, Sophie Nordahl Rasmussen, Lone Baandrup, Line Malmer Madsen, Birte Yding Glenthøj, Egill Rostrup, Brian Villumsen Broberg

**Affiliations:** ^1^Centre for Neuropsychiatric Schizophrenia Research (CNSR), Centre for Clinical Intervention and Neuropsychiatric Schizophrenia Research (CINS), Mental Health Centre Glostrup, University of Copenhagen, Glostrup, Denmark; ^2^Department of Clinical Medicine, Faculty of Health and Medical Sciences, University of Copenhagen, Copenhagen, Denmark; ^3^Functional Imaging Unit, Department of Clinical Physiology and Nuclear Medicine, Rigshospitalet Glostrup, University of Copenhagen, Copenhagen, Denmark; ^4^Department of Anaesthesia, Glostrup Hospital, University of Copenhagen, Glostrup, Denmark

**Keywords:** glutamate, magnetic resonance spectroscopy, cerebral blood flow, pseudo-continuous arterial spin labelling, ketamine, schizophrenia, structural brain changes

## Abstract

Progressive loss of brain tissue is seen in some patients with schizophrenia and might be caused by increased levels of glutamate and resting cerebral blood flow (rCBF) alterations. Animal studies suggest that the normalisation of glutamate levels decreases rCBF and prevents structural changes in hippocampus. However, the relationship between glutamate and rCBF in anterior cingulate cortex (ACC) of humans has not been studied in the absence of antipsychotics and illness chronicity. Ketamine is a noncompetitive *N*-methyl-D-aspartate receptor antagonist that transiently induces schizophrenia-like symptoms and neurobiological disturbances in healthy volunteers (HVs). Here, we used S-ketamine challenge to assess if glutamate levels were associated with rCBF in ACC in 25 male HVs. Second, we explored if S-ketamine changed the neural activity as reflected by rCBF alterations in thalamus (Thal) and accumbens that are connected with ACC. Glutamatergic metabolites were measured in ACC with magnetic resonance (MR) spectroscopy and whole-brain rCBF with pseudo-continuous arterial spin labelling on a 3-T MR scanner before, during, and after infusion of S-ketamine (total dose 0.375 mg/kg). In ACC, glutamate levels were associated with rCBF before (*p* < 0.05) and immediately following S-ketamine infusion (*p* = 0.03), but not during and after. S-Ketamine increased rCBF in ACC (*p* < 0.001) but not the levels of glutamate (*p* = 0.96). In subcortical regions, S-ketamine altered rCBF in left Thal (*p* = 0.03). Our results suggest that glutamate levels in ACC are associated with rCBF at rest and in the initial phase of an increase. Furthermore, S-ketamine challenge transiently induces abnormal activation of ACC and left Thal that both are implicated in the pathophysiology of schizophrenia. Future longitudinal studies should investigate if increased glutamate and rCBF are related to the progressive loss of brain tissue in initially first-episode patients.

## Introduction

Schizophrenia is a devastating disease with a progressive loss of brain tissue in a subgroup of patients (1). The cause of the loss is currently unknown, but persistently high levels of the neurotransmitter glutamate and alterations of resting cerebral blood flow (rCBF) might be implicated. The loss of brain tissue is among others seen in the temporal and frontal regions (1–5) comprising the anterior cingulate cortex (ACC) and hippocampus that both might be implicated in the pathophysiology of schizophrenia (6–9). Interestingly, increased brain glutamate in rodents has been linked to structural changes in ACC and hippocampus (10, 11). In addition, preclinical studies suggest that glutamate is a key regulator of rCBF (12). However, studies investigating the association between glutamate, rCBF, and structural changes in patients with schizophrenia are sparse and have mainly focused on the hippocampus. In hippocampus of unmedicated patients, a negative association between glutamatergic metabolites and brain volume has been found (13), and in prodromal patients, a correlation between cerebral blood volume and structural brain changes in patients that later transitioned to psychosis was observed (11). Interestingly, a rodent study found that the normalisation of increased brain glutamate in hippocampus was able to both normalize rCBF and prevent structural changes (11). This suggests that glutamate-modulating agents might be neuroprotective, at least in hippocampus. However, the association between glutamate, rCBF, and structural changes has not been explored in ACC and nearby prefrontal areas where increased glutamatergic metabolites are found in some studies of early schizophrenia (14–16), and the loss of brain tissue is seen both early and later in the illness (1–5). Only one study has examined the association between glutamate and rCBF in ACC of medicated patients (17). This study found a positive correlation between rCBF in white matter (WM) and levels of glutamate in a large group of patients, but interpretation was limited by treatment with antipsychotics and a broad age range since both factors affect glutamate (18, 19) and rCBF (20). The confounding effects of antipsychotics and illness duration can be avoided by recruiting first-episode, antipsychotic-naïve or minimally treated patients, but glutamate and rCBF have only been studied separately in this patient group. Glutamatergic metabolites in ACC or nearby medial prefrontal cortex (mPFC) are either increased (14–16), decreased (21), or similar (22) compared to healthy volunteers (HVs). Likewise, rCBF studies have found increased (23, 24), decreased (25), and unchanged (26, 27) levels in the prefrontal cortex of antipsychotic-naïve schizophrenia. Although speculatory, these findings might reflect that a subgroup of patients is characterized by both increased glutamatergic activity and enhanced rCBF. This could very well be the subgroup, where progressive loss of brain tissue is found later in the illness (1). In sum, studies investigating the association between glutamate and rCBF in ACC of antipsychotic-naïve schizophrenia are warranted given that the normalisation of these disturbances could prevent structural changes in hippocampus. Early prevention of progressive loss of brain tissue in the course of schizophrenia is clinically relevant because structural changes have been associated with poorer functional outcome (28).

Pharmacological models of schizophrenia are an alternative approach to study brain abnormalities not confounded by antipsychotics and illness duration. Ketamine is a noncompetitive N-Methyl-D-Aspartate receptor antagonist that transiently induces schizophrenia-like symptoms when administered to HV (29, 30). Ketamine also increases glutamate in the prefrontal cortex of rats (31) and glutamate, glutamine (gln), or gln/glutamate in ACC of HVs in some (32–34), but not all studies (35). Ketamine also enhances rCBF in ACC and other prefrontal areas in HV (36–39). The sub-anaesthetic doses used correspond to doses used to treat depressive disorder (40), and ketamine challenge is generally considered safe.

The primary aim of this study was to investigate if glutamate and rCBF in ACC were associated before, during, and after the infusion of a sub-anaesthetic dose of S-ketamine administered to HVs. Because abnormal thalamocortical interactions might underlie schizophrenia (41) and lead to striatal, dopaminergic disturbances (8, 42–44), we also examined rCBF alterations during S-ketamine infusion in the accumbens and thalamus (Thal) that are connected with ACC (45).

## Participants and Methods

The study was a noncontrolled pre–post intervention design where 25 nonsmoking, right-handed healthy male volunteers aged 21–31 years received constant i.v. infusion of S-ketamine (Pfizer) during magnetic resonance imaging (MRI) scanning. The dosing regimen was 0.25 mg/kg for 20 min and thereafter 0.125 mg/kg for 20 min to keep blood levels stable. S-Ketamine was used since the racemic form (mixture of S- and R-ketamine) is not available in Denmark.

**Figure 1:**
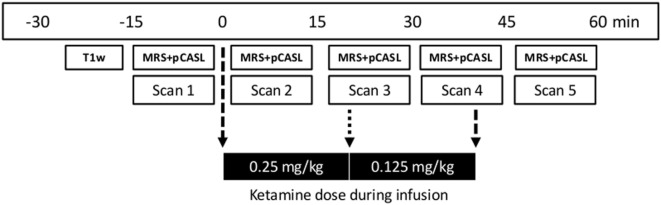
Time line of magnetic resonance imaging acquisitions and dose regimen of S-ketamine infusion. First, a T1-weighted structural scan (T1w; duration 10 min) was acquired followed by five sets of ^1^H-MRS and pCASL data (total acquisition time 11 min) before (scan 1), during (scans 2–4), and after (scan 5) S-ketamine infusion. Stippled arrows indicate the start and end of infusion and the dotted arrow dose change. MRS, Magnetic resonance spectroscopy; pCASL, pseudo-Continuous Arterial Spin Labelling.

Levels of glutamate in ACC and whole-brain rCBF were obtained before, during, and after S-ketamine infusion as shown in Figure 1.

Exclusion criteria were current or previous psychiatric illness tested with Schedules for Clinical Assessment in Neuropsychiatry (46), drug and alcohol abuse as reported by self-report and confirmed with urine testing (Rapid Response, Jepsen HealthCare, Tune, DK), psychiatric disease in first-degree relatives, past or present physical illness, the use of nicotine substitutes, the previous use of ketamine; or the use of benzodiazepines, antipsychotics, anticonvulsants, or antidepressant (as e.g., sleeping medication) within the past 2 months. The study was approved by the Committee on Biomedical Research Ethics for the Capital Region of Denmark (H-4-2014-033), and all participants provided written informed consent after the study procedures were fully explained.

Psychotomimetic effects were assessed with the Positive and Negative Syndrome Scale (PANSS) (47) and the effect on mood with the Positive and Negative Affect Schedule (PANAS) (48) by trained raters before and after S-ketamine infusion. For assessments after infusion, participants were asked about their experiences during infusion while being on the scanner.

### Magnetic Resonance Acquisitions

Data were acquired using a 3-T Philips Achieva system (Philips Healthcare, Eindhoven, Netherlands) equipped with a 32-channel head coil (*Invivo*, Orlando, FL, USA). A whole-brain three-dimensional high-resolution T1-weighted structural scan (TR 10 ms, TE 4.6 ms, flip angle = 8°, and voxel size = 0.79 mm × 0.79 mm × 0.80 mm) was obtained for grey matter (GM) and white matter (WM) WM tissue classification and anatomical reference. A forehead strap was placed to minimize head motion. One participant moved approximately 2 mm, all others <0.7 mm.

**Figure 2:**
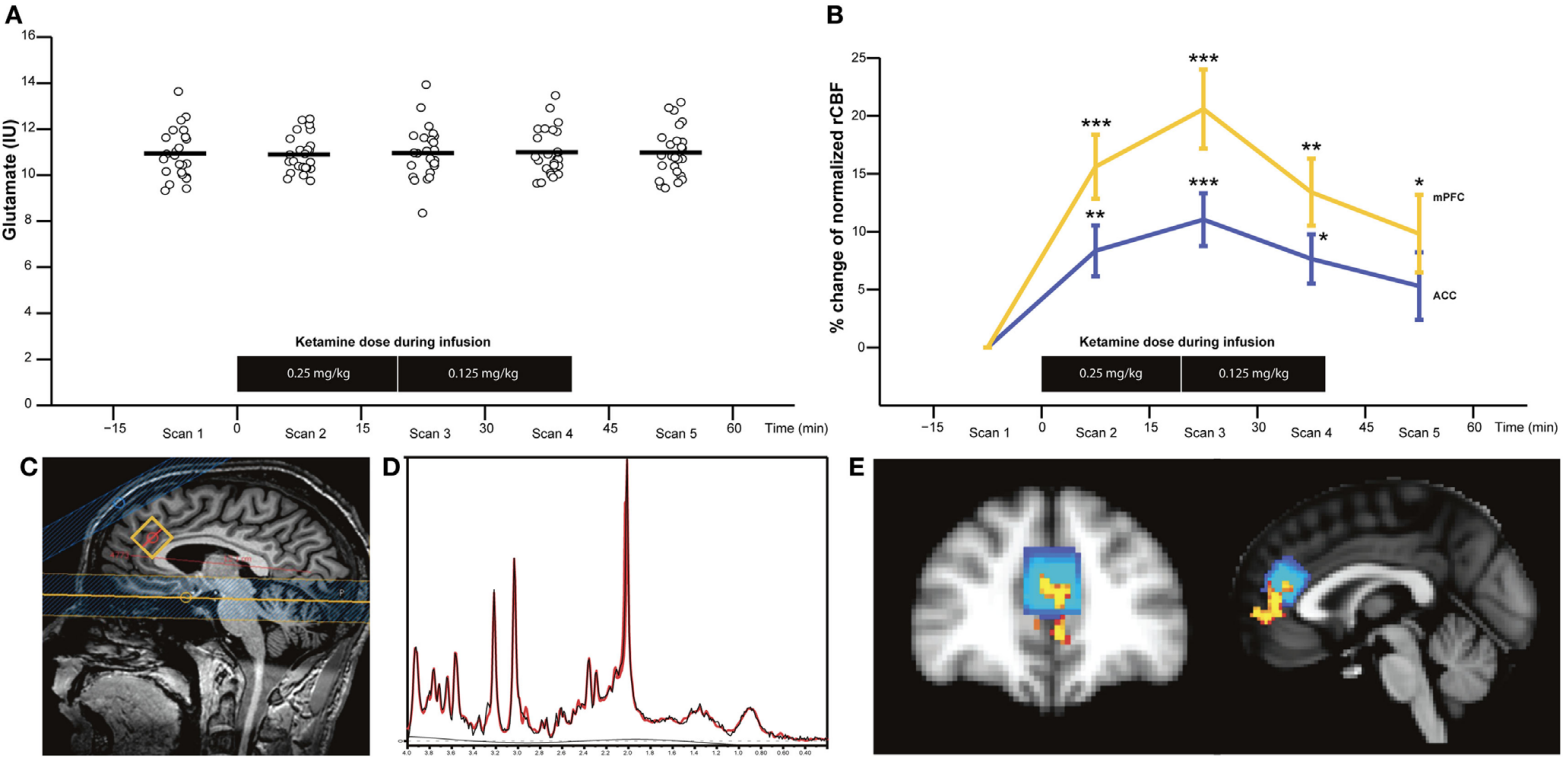
Glutamate levels in anterior cingulate cortex (ACC) (*n* = 25) **(A)** and percentage change of normalised resting cerebral blood flow (rCBF) in ACC and medial prefrontal cortex (mPFC) (*n* = 16) **(B)** before (scan 1), during (scans 2–4), and after S-ketamine infusion (scan 5). Sagittal image with proton magnetic resonance spectroscopy voxel location **(C)**, representative spectra with raw data (in black) and fitted data (in red) **(D)**, and image of the overlap between the area with the most significant rCBF increase corresponding to mPFC (in yellow) and the ACC corresponding to the MRS voxel (blue) are shown **(E)**. Horizontal bars represent the mean values. *p < 0.0125, **p < 0.0025, and ***p < 0.00025 (*p* < 0.05/4 to control for multiple comparisons).

Proton magnetic resonance spectroscopy (^1^H-MRS) and unsuppressed water reference spectra were obtained with frequency-stabilised point-resolved spectroscopy (TE 30 ms, TR 3,000 ms, 128 averages with MOIST water suppression). A 2.0 cm × 2.0 cm × 2.0 cm voxel was prescribed in ACC (Brodmann areas 24 and 32) by drawing a line through the extremities of corpus callosum, placing the point of the voxel at the intersection and aligning to corpus callosum as shown in Figure 2C. Similar acquired acquisitions have revealed good test–retest reliability for glutamate with a percentage coefficient of variation <7% (unpublished data). Acquisition time was 7 min.

A pseudo-Continuous Arterial Spin Labelling (pCASL) sequence was used to assess rCBF as described elsewhere (49). The sequence consisted of 30 pairs of perfusion weighted and control scans (dual echo EPI; 16 slices of 5 mm with an in-plane resolution of 3.55 mm × 3.55 mm; SENSE factor 2.3; TR = 4,100 ms; TE = 12 ms, 28.5 ms at a post-labelling delay of 1,600 ms; labelling duration 1,650 ms; background inversion pulses at 1,663 and 2,850 ms after the start of labelling). *M*0 scan: TR/TE = 10 s/9 ms. Acquisition time was 4 min.

### ^1^H-MRS Analysis

Proton magnetic resonance spectroscopy spectra were analysed with LCModel version 6.3-1 J (50) within the spectral range of 0.2 and 4.0 ppm using water scaling to estimate the concentration of neurometabolites from a standard basis set comprising alanine, aspartate, creatine (Cr), phosphocreatine (PCr), GABA, glucose, gln, glutamate, glycerophosphocholine (GPC), phosphocholine (PCh), glutathione, *myo*-inositol (Ins), lactate, N-acetyl aspartate (NAA), NAA glutamate, *scyllo*-Ins, and taurine. Spectra quality was evaluated by visual inspection, and individual neurometabolites with Cramer–Rao Lower bound (CRLB) >20% were excluded. The percentage of GM and WM in the ^1^H-MRS voxel was estimated and used to calculate institutional units of glutamate (glu_IU_), glx (glx_IU_), and gln_IU_ corrected for cerebrospinal fluid contamination as described in the Supplementary Material. Glu_IU_ was used as the primary outcome, and glx_IU_ and gln_IU_ were analysed as the secondary outcomes to allow comparison with other studies. In exploratory analyses, glutamate, glx, and gln scaled to Cr (Cr + PCr) were analysed.

### pCASL Analysis

Calculation of rCBF was done using the FSL software package (https://fsl.fmrib.ox.ac.uk/fsldownloads_registration). First, the “Brain extraction Tool” was used to remove non-brain tissue from a T1-weighted image. Second, the pCASL data obtained before, during, and after ketamine infusion were co-registered with the skull-stripped T1-weighted image, and, lastly, the T1-weigthed image was nonlinearly co-registered to Montreal Neurological Institute (MNI) space and the combined transformation was applied to the rCBF maps.

The effects of S-ketamine on rCBF were investigated with both voxel-based and region of interest (ROI) analyses. Voxel-based analysis identified areas with the most significant increase of rCBF using an analysis of variance (ANOVA) model and permutation-based statistical inference. We further used threshold-free cluster enhancement to account for spatial dependencies. The statistical maps were thresholded at *p* < 0.05 and corrected for multiple comparisons (FWE correction). ROI analyses were performed in two defined cortical areas with the first corresponding to the position of the MRS voxel in ACC and the second to the region with most significant voxel-based changes of rCBF, and in the subcortical regions provided by the MNI atlas from FSL (left and right Thal, caudate, accumbens, and putamen). The rCBF was calculated as both absolute values in mL/100 g/min and as normalised values by dividing each voxel with the global mean for each subject to reduce inter-subject variation caused by a difference in global rCBF. Normalised rCBF was used as the primary outcome to enhance the sensitivity to regional changes, but absolute rCBF values are reported as well.

### Statistics

The primary hypothesis that levels of glutamate (independent variable) would be associated with normalised rCBF in ACC (dependent variable) during each of the five scans was tested with five separate linear regression models with a significance level set to *p* < 0.05 for this *a priori* hypothesis.

The main effect of S-ketamine on levels of glutamatergic metabolites and rCBF was evaluated separately using a one-way repeated measures ANOVA (rmANOVA) with statistical significance defined as *p* < 0.05 for the main effects and *p* < 0.0125 for *post hoc t*-tests (separate *t*-tests Bonferroni corrected with *p*/4 scans during S-ketamine infusion). Multivariate tests of the main effect are reported if the assumption of sphericity was violated. No outliers were identified according to Cook distance criterion (51).

PANSS (total, positive, negative, and general subscores) and PANAS (positive- and negative-affect scores) before and after ketamine infusion were analysed using Wilcoxon signed rank test. Correlations between mental state effect changes induced by S-ketamine and levels of glutamate or normalised rCBF in the ACC voxel were tested with Spearman’s rho and corrected for multiple comparisons (Bonferroni). Statistical analyses were performed in SAS version 7.1 (SAS institute, Cary, NC, USA).

## Results

### Mental State Changes and Laboratory Results with S-Ketamine

The mental state changes with S-ketamine and demographic variables of participants are summarized in Table 1. S-Ketamine significantly increased PANSS total, and all subscores with items P2 (disorganized thinking) and P3 (hallucinations) being most prominently affected with an increase of 104 and 184%, respectively. Furthermore, S-ketamine significantly decreased positive affect. The negative affect increased but not to a significant extent.

**Table 1:** Demographic characteristics and psychotomimetic effects with S-ketamine.

Characteristic		Means
*N* (males only)		25
Age, years ± SD		25.4 ± 3.3
BMI, kg/m^2^ ± SD		23.5 ± 2.0
Years of education ± SD		14 ± 2
PANSS total ± SEM	Pre	32.0 ± 0.5
	Post	38.4 ± 1.2***
PANSS positive ± SEM	Pre	7.5 ± 0.3
	Post	10.8 ± 0.4***
PANSS negative ± SEM	Pre	7.5 ± 0.1
	Post	8.5 ± 0.5**
PANSS general ± SEM	Pre	17.0 ± 0.3
	Post	19.2 ± 0.7**
Positive affect ± SEM	Pre	30.7 ± 1.2
	Post	23.5 ± 1.5***
Negative affect ± SEM	Pre	11.8 ± 0.4
	Post	13.3 ± 0.7

*BMI, body mass index; PANSS, Positive and Negative Syndrome Scale*.

**p < 0.01.

***p < 0.001.

Serum levels of S-ketamine obtained from four HVs after scan 5 as test samples were 140.5 ± 27.0 ng/mL.

### Glutamatergic Metabolites before, during, and after S-Ketamine

No spectra were excluded after visual inspection, and the quality was good as shown in a representative spectrum in Figure 2D. CRLB values were <9% for glutamate and glx, but 23 gln-values were excluded due to CRLB >20%. Full-width half-maximum, signal-to-noise ratio, and CRLB for glutamate, NAA, myo-Ins, and choline did not differ during the five MRS acquisitions, but CRLB for glx, gln, and PCr + Cr did as summarized in Table S1 in Supplementary Material.

*Glu_IU_, glx_IU_, gln_IU_, and other neurometabolites*: There were no significant main effects of time for glu_IU_ [*F*(4, 96) = 0.13, *p* = 0.962] (Figure 2A), glx_IU_ [*F*(4, 96) = 1.07, *p* = 0.374], gln_IU_ [*F*(4, 36) = 0.64, *p* = 0.635], or other neurometabolites such as NAA, Cr + PCr, choline (GPC + PCh), and myo-Ins. The mean values of metabolites are provided in Table S2 in Supplementary Material. The inclusion of glutamate, glx, or gln scaled to Cr in the rmANOVA did not change the results. Lastly, no main effect of time was found for glu_IU_, glx_IU_, and gln_IU_ in the subgroup of HV, which also had a pCASL scan (*n* = 16). In sum, S-ketamine infusion did not appear to affect glutamatergic metabolites in the ACC voxel.

### rCBF before, during, and after S-Ketamine

pseudo-Continuous Arterial Spin Labelling data were only usable for 16 subjects due to technical challenges.

#### Voxel-Based Analysis

**Figure 3:**
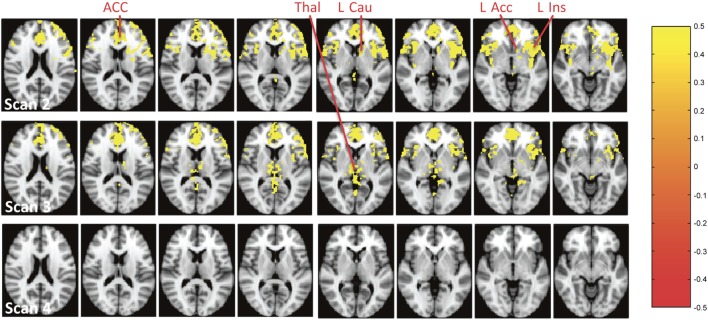
Brain regions with a significant increase of absolute resting cerebral blood flow (mL/100 g/min) during the infusion of S-ketamine at scans 2–4. The colours represent *p*-values from the voxel-based analysis as shown to the right. Abbreviations: ACC, anterior cingulate cortex; Thal, thalamus; l Cau, left caudate (ventral part); L Acc, left accumbens; l Ins, left insula.

Voxel-based analysis of the absolute rCBF (mL/100 g/min) revealed that the most significant increase of rCBF during scan 2 was in mPFC/ACC, insula, left accumbens (L Acc), and left ventral caudate (Figure 3, scan 2), and during scan 3 in left and right Thal (Figure 3, scan 3).

After normalizing rCBF, the most significant increase was seen in Brodmann area 32 (MNI coordinates *x* = 45, *y* = 83, *z* = 45) that corresponds to mPFC and partly overlaps with the MRS voxel in ACC (Figure 2E).

#### ROI Analyses of Absolute and Normalised rCBF Changes

The rmANOVA of ROIs revealed a significant main effect of time for absolute rCBF (mL/100 g/min) in the two defined cortical ROIs mPFC (*F*(4, 60) = 29.6, *p* < 0.0001) and the ACC voxel (denoted ACC hereinafter) (*F*(4, 60) = 13.44, *p* = 0.0002) with *post hoc* tests revealing a significant increase in mPFC during scan 2 (26 ± 3%, *p* < 0.0001), scan 3 (30 ± 4%, *p* < 0.0001), scan 4 (21 ± 5%, *p* = 0.0014), and at trend level that did not survive correction for multiple comparisons during scan 5 (12 ± 4%, *p* = 0.025), and in ACC during scan 2 (18 ± 3%, *p* < 0.0001), scan 3 (20 ± 3%, *p* = 0.0001), and scan 4 (15 ± 4%, *p* = 0.006), but not during scan 5 (7 ± 4%, *p* = 0.11). Absolute values in mL/100 g/min for the five scans are reported in Table S3 in Supplementary Material. In the subcortical ROIs, a significant main effect of time was seen in left and right Thal, left caudate (L Cau), and L Acc, although the *post hoc* tests only were significant during scan 2 for L Cau and L Acc as reported in Table S4 in Supplementary Material.

After normalizing rCBF, the main effect of S-ketamine remained significant in mPFC (*F*(4, 60) = 15.06, *p* < 0.0001), ACC (*F*(4, 60) = 5.45, *p* = 0.0008), and left Thal (*F*(4, 12) = 4.08, *p* = 0.026). *Post hoc* tests revealed a significant increase in mPFC and ACC during scans 2–4, and additionally in mPFC during scan 5 (Figure 2B). In left Thal, normalised rCBF appeared to decrease during scan 2; however, the *post hoc* tests were insignificant. No main effect of time was seen in right Thal, 1 Cau, right caudate, right accumbens, left putamen, and right putamen. Statistics and percentage increase compared to preinfusion (scan 1) of normalised rCBF values for cortical and subcortical ROIs are provided in Tables S5 and S6 in Supplementary Material, respectively.

In sum, S-ketamine infusion affected mPFC/ACC and left Thal after correction for the effect of global blood flow (normalisation).

### Relationship between Levels of Glutamate and Normalised rCBF in ACC before, during, and after S-Ketamine

**Figure 4:**
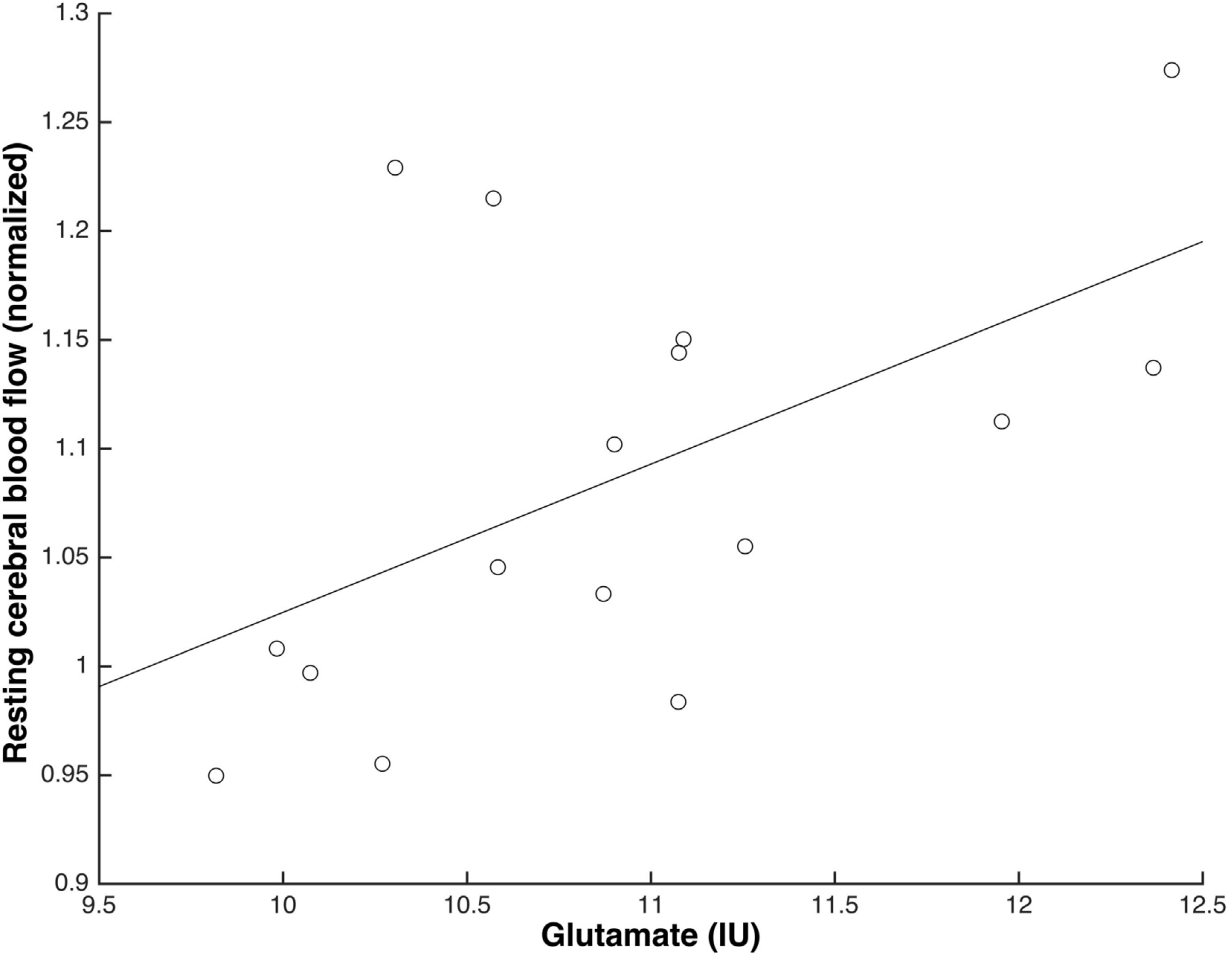
Glutamate levels and normalised resting cerebral blood flow (rCBF) in anterior cingulate cortex were positively associated immediately following S-ketamine infusion (scan 2) (*N* = 16; *b* = 0.07, *t* = 2.41, *p* = 0.03).

Higher levels of glu_IU_ were significantly associated with higher values of normalised rCBF in ACC prior to S-ketamine infusion during scan 1 (*b* = 0.05, *t* = 2.19, *p* = 0.046) and immediately following infusion during scan 2 (*b* = 0.07, *t* = 2.41, *p* = 0.03, Figure 4), but not to a significant extent during scan 3 (*b* = 0.04, *t* = 1.64, *p* = 0.12), scan 4 (*b* = 0.01, *t* = 0.45, *p* = 0.66), or scan 5 (*b* = 0.02, *t* = 0.62, *p* = 0.55). When adjusting for age, similar results were observed, although only borderline significant during scan 1 (scan 1: *p* = 0.05; scan 2: *p* < 0.05; scan 3: *p* = 0.15; scan 4: *p* = 0.60; scan 5: *p* = 0.56).

Glx_IU_ was associated with normalised rCBF in ACC prior to S-ketamine infusion at a trend level that did not survive correction for multiple comparison (*p* < 0.05/2 = 0.025) during scan 1 (*b* = 0.03, *t* = 2.29, *p* = 0.038), and significantly during scan 2 (*b* = 0.06, *t* = 3.85, *p* = 0.002) but not during scans 3, 4, or 5 (*p* > 0.05). Similar results were obtained when adjusting for age (scan 1: *p* = 0.035; scan 2: *p* = 0.002; scans 3–5: *p* > 0.05).

No association was found between gln_IU_ and normalised rCBF in ACC, neither when adjusting for age. Also, there was no main effect of age for any of the metabolites, and all the glu_IU_/glx_IU_/gln_IU_ × age interactions were insignificant and removed from the analyses.

### Relationship between Changes in Mental State with S-Ketamine, Levels of Glutamate, and Normalised rCBF in mPFC

Neither glutamate in ACC nor normalised rCBF in mPFC during S-ketamine infusion (scans 2–4) correlated with changes in PANSS total, positive, negative, and general or positive and negative affect to a significant extent.

### Physiological Data

Ketamine infusion did not significantly affect blood pressure (systolic) or heart rate.

## Discussion

The primary finding of this study was that levels of glutamate were positively associated with normalised rCBF in ACC before and immediately following the infusion of S-ketamine in HV. In addition, S-ketamine transiently induced abnormal neural activation as measured by altered normalised rCBF in mPFC, ACC, and left Thal. However, levels of glutamate in ACC were not affected during S-ketamine infusion.

The results support that glutamate levels and rCBF are associated in ACC as also seen in a recent study of medicated patients with schizophrenia (17), a rodent study of hippocampus (11), and preclinical studies (12). Importantly, this association was independent of antipsychotic exposure and psychiatric illness chronicity.

In terms of clinical relevance, a rodent study revealed that the normalisation of increased glutamate and enhanced rCBF could prevent structural changes in hippocampus (11). Given that glutamate and rCBF were associated in ACC in our study, it is likely that glutamate-modulating agents also might be neuroprotective in this area as well when given to schizophrenia patients with increased glutamate levels. The progressive loss of brain tissue in schizophrenia is only seen in a subgroup of patients (1), and we speculate if increased glutamatergic activity and enhanced rCBF characterize the subgroup of patients who experience structural changes later in the illness. Thus, future studies should aim at investigating if increased glutamate in ACC and enhanced rCBF in first-episode patients predict the progressive loss of brain tissue.

The association between glutamate levels and normalised rCBF in the ACC was only seen before (scan 1) and immediately following (scan 2), but not during (scans 3 and 4) or after (scan 5) S-ketamine infusion, which can indicate that glutamate mainly regulates rCBF during an increase in HVs, whereas other factors might be involved during the maintenance and decrease of enhanced rCBF. In preclinical studies, glutamatergic neurotransmission plays a key role in the regulation of cerebral blood flow by activating N-Methyl-D-Aspartate receptors on neurons and metabotropic glutamate receptors on astrocytes (12). The subsequent rise in intracellular Ca^2+^ leads to the release of intracellular-vasodilating messengers but can also cause blood vessel constriction (12). The outcome of intracellular Ca^2+^ rise is influenced by preexisting vessel tone and the O_2_ concentration, in that dilation occurs with physiological O_2_ concentrations and constriction with supraphysiological O_2_ concentrations (12, 52). Although speculative, our findings might imply that S-ketamine initially induces vasodilation, but that this effect diminishes or ceases after some time due to persistently increased O_2_ and enhanced vessel tone.

Levels of glutamate as measured with ^1^H-MRS in ACC were unaffected during the infusion of S-ketamine. This is in agreement with one previous study (35) but in contrast with two others (32, 33). Several factors might explain this finding. First, the ^1^H-MRS voxel in ACC was placed a bit dorsal from mPFC where the most significant increase of rCBF was seen (Figure 2E). Although the ACC voxel and mPFC overlap, the increase of glutamate levels in ACC might not have been sufficient to be detected with ^1^H-MRS. Second, our dose regimen of S-ketamine might have been too high. We administered pure S-ketamine in a dosing regimen corresponding to previous studies where the racemic form was used (50% R- and 50% S-ketamine) (32, 33, 35), but it seems likely that the dose of pure S-ketamine and the racemic form does not correspond to 1:1 since R-ketamine restricts the clearance of the S-enantiomer (53). In addition, racemic ketamine mainly increases extracellular cortical glutamate at low doses in rats (31), and it is notable that the two ^1^H-MRS studies that found increased levels of glutamate (33) or gln (32) used a lower dose of racemic ketamine than in the present study and the other study where glutamate was unaffected (35). Third, one of the MRS ketamine studies found increased gln but not glutamate (32). Gln reflects glutamate released by the synapse and taken up by astrocytes only (54), whereas glutamate reflects other metabolic processes as well (55). Therefore, minor changes of the glutamate level in the synaptic cleft can be blurred by the glutamate contained in presynaptic vesicles and astrocytes. However, gln is challenging to accurately quantify at field strengths below 4 T due to overlapping resonance frequencies with glutamate. Lastly, a ketamine-induced increase of glutamatergic metabolites might be easier to detect in pathological conditions like depression, where resting glutamate is decreased (56).

S-Ketamine significantly affected rCBF in several areas. The voxel-based analysis revealed that the most significant increase of both absolute (mL/100 g/min) and normalised rCBFs was in ACC and the overlapping mPFC (Figures 2B,E), which is in line with previous PET (36, 37) and MRI studies (39), administering racemic ketamine to HVs. This confirms that the main effect of S-ketamine also is mediated through ACC/mPFC. The rCBF enhancements are most likely induced by the S-enantiomer, since the administration of pure R-ketamine seems to decrease rCBF in HVs (57).

Our second aim was to explore if S-ketamine transiently induced the abnormal neural activation of the accumbens and Thal as reflected by rCBF alterations since these areas are connected with ACC (45) and implicated in the pathophysiology of schizophrenia (8, 42–44). Absolute rCBF was significantly increased in L Acc and left ventral caudate (left ventral striatum) and Thal (Figure 3, scan 2). However, only the effect in ACC/mPFC remained significant in the voxel-based analyses when rCBF was normalised. In the ROI analyses, a significant increase of absolute rCBF was also seen in ACC, mPFC, left and right Thal, L Acc, and L Cau (Tables S3 and S4 in Supplementary Material) but only remained significant in ACC, mPFC, and left Thal after normalizing rCBF (Tables S5 and S6 in Supplementary Material). In left Thal, S-ketamine seemed to decrease normalised rCBF during scan 2, although not to a significant extent. This decrease might reflect a feedback mechanism due to the increased activity of ACC and mPFC. In sum, S-ketamine mainly seems to affect ACC/mPFC and left Thal, although accumbens might be activated to a minor extent. This is in accord with a ketamine study of rats that found mainly increased cortical glutamate and only to a minor extent increased striatal dopamine (31), and a SPECT study of HVs where striatal dopamine was unaffected by ketamine (58). Taken together, it seems that ketamine challenge only affects striatal dopaminergic activity to a minor extent. This is a limitation if ketamine challenge is used as a pharmacological model of schizophrenia where increased striatal dopaminergic activity is one of the best validated findings (59). Interestingly, the SPECT study found that striatal dopaminergic activity in HV was enhanced more by administering both amphetamine and ketamine than amphetamine alone (58). It is possible that this combined administration mimics the neurobiology of schizophrenia better.

S-Ketamine transiently induced schizophrenia-like symptoms as measured with PANSS and thereby replicated previous findings (29, 30). PANSS positive increased by three points, which in general is considered clinically relevant if the impact of novel treatments is to be tested during S-ketamine challenge (30). The effect of S-ketamine on mood was measured with PANAS (48) since ketamine has an antidepressant effect as well (40). In contrast to this, we found that S-ketamine significantly decreased positive affect, which might reflect the negative symptoms induced by S-ketamine in this and other studies (29, 30). In addition, a recent study of mice indicates that only the R-enantiomer has an antidepressant effect (60).

Previous PET studies have reported a positive correlation between enhanced rCBF in the ACC and schizophrenia-like symptoms, mainly psychosis (36–38). By contrast, we did not find any significant correlations between the maximal rCBF increase in mPFC and the mental state effects induced by S-ketamine. Interestingly, this is in line with another recent pCASL ketamine challenge study (39). Several factors might influence the inconsistency between studies. First, the use of PET versus MR can impact findings. Second, different dosing regimens were used, and the psychotomimetic effects of ketamine are dose-dependent (30). Third, S- and R-ketamine induce differential psychopathology (57) and differences can be ascribed to the use of racemic versus S-ketamine. Moreover, different rating scales were used. Lastly, our data might have lacked power since pCASL data only were available for 16 subjects out of 25.

The study has several limitations that should be mentioned. First, we did not include a placebo group and cannot rule out that levels of glutamate increased preinfusion due to expectation of S-ketamine administration. Second, we did not include any scales to assess the dissociation effect of S-ketamine. Lastly, only 16 of the subjects had a useable pCASL scan, and some analyses might be underpowered.

In conclusion, our findings support the notion that glutamate levels in ACC are associated with rCBF in male HVs at rest and during the initial phase of an increase, and that S-ketamine transiently induces psychotomimetic effects and alters the activation of the ACC/mPFC and left Thal, resembling important aspects of schizophrenia. Future S-ketamine challenge studies can be improved by including a placebo group, placing the ^1^H-MRS voxel more ventral, and optimising the dose regimen of S-ketamine for measures of glutamatergic metabolites. Furthermore, studies investigating the association between prefrontal glutamate, rCBF, and progressive loss of brain tissue in initially first-episode patients with schizophrenia are warranted.

## Ethics Statement

This study was carried out in accordance with the recommendations of “guidelines for research with legally competent adults, Danish National Committee on Biomedical Research Ethics” with written informed consent from all subjects. All subjects gave written informed consent in accordance with the Declaration of Helsinki. The protocol was approved by the “Committee on Biomedical Research Ethics for the Capital Region of Denmark (H-4-2014-033).”

## Author Contributions

All authors made substantial contributions to all of the following: (1) the conception and design of the study, or acquisition of data, or analysis and interpretation of data, (2) drafting the article or revising it critically for important intellectual content, (3) final approval of the version to be submitted, and (4) all authors agree to be accountable for all aspects of the work. Specifically, KB, KA, LB, ER, LM, BB, and BG designed the study; KA, SN, and BB collected the data; and KB, KA, SN, BB, and ER analysed the data. KB, KA, and BB drafted the manuscript.

## Conflict of Interest Statement

BG is the leader of a Lundbeck Foundation Centre of Excellence for Clinical Intervention and Neuropsychiatric Schizophrenia Research (CINS), which is partially financed by two independent grants from the Lundbeck Foundation (R25-A2701 and R155-2013-16337), and partially financed by the Mental Health Services in the Capital Region of Denmark, the University of Copenhagen, and other foundations. All grants are the property of the Mental Health Services in the Capital Region of Denmark and administrated by them. KB, KA, SR, LM, ER, and BB declare no potential conflict of interest.
