# Supplementary material for: Glutamate Levels and Resting Cerebral Blood Flow in Anterior Cingulate Cortex Are Associated at Rest and Immediately Following Infusion of S-Ketamine in Healthy Volunteers
Source: Front Psychiatry. 2018 Feb 6;9:22. doi: 10.3389/fpsyt.2018.00022 (PMC5808203; doi:10.3389/fpsyt.2018.00022)
Supplement: Supplementary file 1 [file Data_Sheet_1.docx]

Supplementary Material

**Glutamate levels and resting cerebral blood flow in anterior cingulate cortex are associated at rest and immediately following infusion of S-ketamine in healthy volunteers**

Authors: Kirsten Borup Bojesen, MD^*^; Kasper Aagaard Andersen, MD; Sophie Nordahl Rasmussen, MD; Lone Baandrup, Ph.D.; Line Malmer Madsen, MD; Birte Yding Glenthøj, DMSc; Egill Rostrup, DMSc; Brian Villumsen Broberg, Ph.D.

*****Corresponding Author: [Kirsten.Borup.Bojesen@regionh.dk](mailto:Kirsten.Borup.Bojesen@regionh.dk)

**Participants and Methods**

*Data analysis*

To estimate the fraction of grey matter (GM) and white matter (WM) in the ^1^H-MRS voxel, the T1-weighted structural image was first segmented using SPM 8 (<http://www.fil.ion.ucl.ac.uk/spm/software/spm8/>) running under MATLAB® (The MathWorks Inc., Massachusetts, USA). This segmentation was combined with the ^1^H-MRS voxel mask and the GM and WM fractions was used with *in vivo* water scaled values of glutamate, glx, and glutamine reported by LCModel to calculate concentrations in institutional units (IU) corrected for cerebrospinal fluid (CSF) contamination using the following equation:

IU_Metabolite_ = M*(WM+GM+1.55*CSF)/(WM+GM)

CSF content in the ^1^H-MRS is calculated as 1-(wm+gm). The equation is described in details in^1^.

**Reference**

1. Stone JM, Dietrich C, Edden R, et al. Ketamine effects on brain GABA and glutamate levels with 1H-MRS: relationship to ketamine-induced psychopathology. *Mol Psychiatry.* 2012;17(7):664-665.
