# Supplementary material for: Glutamate Levels and Resting Cerebral Blood Flow in Anterior Cingulate Cortex Are Associated at Rest and Immediately Following Infusion of S-Ketamine in Healthy Volunteers
Source: Front Psychiatry. 2018 Feb 6;9:22. doi: 10.3389/fpsyt.2018.00022 (PMC5808203; doi:10.3389/fpsyt.2018.00022)
Supplement: Supplementary file 2 [file Table_1.docx]

**Table S1: Spectral quality for ^1^H-MRS acquisitions in anterior cingulate cortex**

|  | **Scan 1** | **Scan 2** | **Scan 3** | **Scan 4** | **Scan 5** | **Statistics^a^ (p)** |
| --- | --- | --- | --- | --- | --- | --- |
|  | **Mean ± SD,**  **n** | **Mean ± SD,**  **n** | **Mean ± SD,**  **n** | **Mean ± SD,**  **n** | **Mean ± SD,**  **n** |  |
| **FWHM** | 0.055 ± 0.020, n=25 | 0.061 ± 0.031,  n=25 | 0.056 ± 0.023,  n=25 | 0.057 ± 0.025,  n=25 | 0.058 ± 0.024,  n=25 | 0.15 |
| **Signal to noise ratio** | 24.8 ± 3.4,  n=25 | 24.2 ± 3.5,  n=25 | 24.2 ± 4.1,  n=25 | 23.6 ± 3.8,  n=25 | 23.8 ± 3.9,  n=25 | 0.33 |
| **CRLB (%) Glutamate** | 5.4 ± 0.9,  n=25 | 5.6 ± 0.8,  n=25 | 5.4 ± 0.7,  n=25 | 5.5 ± 0.7,  n=25 | 5.5 ± 1.0,  n=25 | 0.53 |
| **CRLB (%) Glx** | 5.1 ± 0.7,  n=25 | 5.5 ± 0.9,  n=25 | 5.4 ± 0.9,  n=25 | 5.8 ± 0.8,  n=25 | 5.6 ± 0.8,  n=25 | 0.02 |
| **CRLB (%) Glutamine** | 15.3 ± 2.2,  n=24 | 15.5 ± 1.9,  n=20 | 15.6 ± 2.3,  n=20 | 17.3 ± 2.3,  n=19 | 16.5 ± 2.7,  n=19 | 0.01 |
| **CRLB (%) NAA** | 3.8 ± 1.6,  n=25 | 3.8 ± 1.1,  n=25 | 3.5 ± 1.2,  n=25 | 3.4 ± 1.0,  n=25 | 3.7 ± 1.6,  n=25 | 0.51 |
| **CRLB (%) PCr+Cr** | 3.0 ± 0.5,  n=25 | 3.2 ± 0.5,  n=25 | 3.2 ± 0.5,  n=25 | 3.2 ± 0.5,  n=25 | 3.3 ± 0.7,  n=25 | 0.04 |
| **CRLB (%) Myo-inositol** | 4.7 ± 0.9,  n=25 | 5.0 ± 1.3,  n=25 | 5.0 ± 1.0,  n=25 | 5.2 ± 1.1,  n=25 | 5.0 ± 1.3,  n=25 | 0.10 |
| **CRLB (%) Choline** | 3.4 ± 0.7,  n=25 | 3.9 ± 2.2,  n=25 | 3.4 ± 0.8,  n=25 | 4.2 ± 3.8,  n=24 | 3.5 ± 0.8,  n=25 | 0.53 |

**^a^**Main effect of repeated measure ANOVA
