# Supplementary material for: Glutamate Levels and Resting Cerebral Blood Flow in Anterior Cingulate Cortex Are Associated at Rest and Immediately Following Infusion of S-Ketamine in Healthy Volunteers
Source: Front Psychiatry. 2018 Feb 6;9:22. doi: 10.3389/fpsyt.2018.00022 (PMC5808203; doi:10.3389/fpsyt.2018.00022)
Supplement: Supplementary file 3 [file Table_2.docx]

**Table S2: Mean values of 1H-MRS metabolites before, during, and after S-ketamine infusion**

| **Metabolites** | **Scan 1** | **Scan 2** | **Scan 3** | **Scan 4** | **Scan 5** |
| --- | --- | --- | --- | --- | --- |
|  | **Mean ± SD, n** | **Mean ± SD, n** | **Mean ± SD, n** | **Mean ± SD, n** | **Mean ± SD, n** |
| **Glu_IU_** | 10.92 ± 1.07 , n = 25 | 10.88 ± 0.80, n = 25 | 10.94 ± 1.12, n = 25 | 10.98 ± 1.01, n = 25 | 10.96 ± 1.08 , n = 25 |
| **Glx_IU_** | 15.63 ± 1.60, n = 25 | 15.27 ± 1.38, n = 25 | 15.44 ± 1.57, n = 25 | 15.08 ± 1.40, n = 25 | 15.38 ± 1.47, n = 25 |
| **Gln_IU_** | 4.76 ± 0.86, n = 24 | 4.68 ± 0.77, n = 20 | 4.81 ± 0.83, n = 20 | 4.29 ± 0.48, n = 19 | 4.64 ± 0.88, n = 19 |
| **NAA_IU_** | 8.28 ± 0.85, n = 25 | 8.39 ± 0.80, n = 25 | 8.32 ± 0.98, n = 25 | 8.41 ± 0.82, n = 25 | 8.26 ± 0.83, n = 25 |
| **PCr + Cr^a^** | 5.37 ± 0.28 , n=25 | 5.49 ± 0.42, n=25 | 5.40 ± 0.42, n=25 | 5.46 ± 0.59, n=25 | 5.34 ± 0.36, n=25 |
| **Myo-inositol_IU_** | 5.79 ± 0.60, n=25 | 5.78 ± 0.57, n=25 | 5.61 ± 0.61, n=25 | 5.64 ± 0.66, n=25 | 5.64 ± 0.60, n=25 |
| **Choline_IU_** | 2.00 ± 0.19, n=25 | 2.04 ± 0.29, n=25 | 2.01 ± 0.21, n=25 | 2.03 ± 0.31, n=24 | 2.00 ± 0.25, n=25 |
| ***MRS voxel content*** |  |  |  |  |  |
| **Grey matter (%)** | 70.7 ± 3.9 |  |  |  |  |
| **White matter (%)** | 14.5 ± 2.8 |  |  |  |  |

**^a^** Water scaled values of PCr+Cr as provided in the LCModel output and used as reference for metabolite/Cr values
