# Supplementary material for: Glutamate Levels and Resting Cerebral Blood Flow in Anterior Cingulate Cortex Are Associated at Rest and Immediately Following Infusion of S-Ketamine in Healthy Volunteers
Source: Front Psychiatry. 2018 Feb 6;9:22. doi: 10.3389/fpsyt.2018.00022 (PMC5808203; doi:10.3389/fpsyt.2018.00022)
Supplement: Supplementary file 4 [file Table_3.doc]

**Table S3: Absolute resting cerebral blood flow in medial prefrontal cortex and anterior cingulate cortex**

| **Region of interest** | **Statistics,**  **main effect** | **P,**  **main effect** | **Mean CBF in mL/100g/min, % rCBF increase ± SEM, and post hoc tests ^1^**  **Scan 1 Scan 2 Scan 3 Scan 4 Scan 5** | | | | |
| --- | --- | --- | --- | --- | --- | --- | --- |
| **Medial prefrontal**  **cortex** | F(4, 12) = 29.55^2^ | P<0.0001 | 35.1±2.3  0% | 43.5±2. 5*******  26±3% | 44.7±2.6*******  30±4% | 41.9±2.8******  21±5% | 38.4±1.9  12±4% |
| **Anterior cingulate cortex** | F(4, 12) = 13.44^2^ | P=0.0002 | 30.1±1.8  0% | 35.3±1.9*******  18±3% | 35.8±2.0*******  20±3% | 34.6±2.4*****  15±4% | 32.0±1.6  7±4% |

Changes in absolute resting cerebral blood flow in mPFC and ACC during (scan 2, 3, and 4) and after (scan 5) infusion of S-ketamine compared to pre-infusion (scan 1) (n=16). mPFC corresponds to the area with the most significant increase of rCBF in the voxel-based analysis and ACC corresponds to the ^1^H-MRS voxel. ^1^Statistical significance of post hoc tests defined as: * p<0.0125, **p<0.0025, and ***p<0.00025 (p/ 4 to correct for multiple comparisons)**.** ^2^Multivariate test (Pilai’s trace) due to violation of the assumption of sphericity. mPFC: Medial prefrontal cortex, ACC: Anterior cingulate cortex, SEM: Standart error of the mean, rCBF: Resting cerebral blood flow.
