# Supplementary material for: Glutamate Levels and Resting Cerebral Blood Flow in Anterior Cingulate Cortex Are Associated at Rest and Immediately Following Infusion of S-Ketamine in Healthy Volunteers
Source: Front Psychiatry. 2018 Feb 6;9:22. doi: 10.3389/fpsyt.2018.00022 (PMC5808203; doi:10.3389/fpsyt.2018.00022)
Supplement: Supplementary file 5 [file Table_4.doc]

**Table S4: Absolute resting cerebral blood flow in subcortical regions of interest**

| **Region of interest** | **Statistics,**  **main effect** | **p,**  **main effect** | **Mean CBF in mL/100g/min, % rCBF ± SEM, and post hoc tests^1^**  **Scan 1 Scan 2 Scan 3 Scan 4 Scan 5** | | | | |
| --- | --- | --- | --- | --- | --- | --- | --- |
| **Thalamus, left** | F(4,60)= 3.61 | P= 0.01 | 27.8±1.0  0% | 28.9±0.9  5±2% | 29.9±1.0  9±3% | 29.9±1.0  9±3% | 28.5±0.9  3±3% |
| **Thalamus, right** | F(4,60)= 3.56 | P= 0.01 | 27.7±1.1  0% | 29.5±1.1  7±2% | 29.8±1.1  8±3% | 29.6±1.2  7±4% | 28.1±1.1  2±2% |
| **Caudate, left** | F(4,60)= 3.16 | P= 0.02 | 16. 6±1.1  0% | 18.3±1.2*****  11±3% | 18.1±1.2  10±3% | 18.0±1.4  9±4% | 17.7±1.2  8±3% |
| **Caudate, right** | F(4,60)= 2.33 | P= 0.07 | 16.40±1.25  0% | 17.8±1.5  8±3% | 17.8±1.3  10±3% | 17.2±1.4  6±4% | 17.1±1.3  5±4% |
| **Accumbens, left** | F(4,12)= 8.23**^2^** | P= 0.002 | 25.8±1.8  0% | 29.1±1.7*******  15±3% | 28.5±1.7  13±4% | 28.6±1.7  14±5% | 27.9±1.8  5±1% |
| **Accumbens, right** | F(4,12)=2.85**^2^** | P= 0.07 | 23.6±1.8  0% | 26.7±1.9  15±5% | 26.4±1.9  15±6% | 26.5±2.0  15±6% | 25.5±1.9  11±6% |
| **Putamen, left** | F(4,60)= 2.18 | P= 0.08 | 22.8±1.3  0% | 24.8±1.4  9±3% | 24.0±1.4  6±3% | 24.0±1.4  6±3% | 23.0±1.4  4±3% |
| **Putamen, right** | F(4,12)= 3.18**^2^** | P= 0.05 | 21.5±1.5  0% | 23.1±1.5  8±2% | 22.9±1.5  8±3% | 22.8±1.5  7±4% | 22.0±1.5  3±3% |

Changes in absolute resting cerebral blood flow in subcortical regions of interest during (scan 2, 3, and 4) and after (scan 5) infusion of S-ketamine compared to pre-infusion (scan 1) (n=16). ^1^Statistical significance of post hoc tests defined as: * p<0.0125, **p<0.0025, and ***p<0.00025 (p/ 4 to correct for multiple comparisons). ^2^Multivariate test (Pilai’s trace) due to violation of the assumption of sphericity. SEM: Standart error of the mean, rCBF: Resting cerebral blood flow.
