# Supplementary material for: Glutamate Levels and Resting Cerebral Blood Flow in Anterior Cingulate Cortex Are Associated at Rest and Immediately Following Infusion of S-Ketamine in Healthy Volunteers
Source: Front Psychiatry. 2018 Feb 6;9:22. doi: 10.3389/fpsyt.2018.00022 (PMC5808203; doi:10.3389/fpsyt.2018.00022)
Supplement: Supplementary file 6 [file Table_5.doc]

**Table S5: Normalized resting cerebral blood flow in medial prefrontal cortex and anterior cingulate cortex**

| **Region of interest** | **Statistics,**  **main effect** | **p,**  **main effect** | **% rCBF increase ± SEM and post hoc tests^1^**  **Scan 1 Scan 2 Scan 3 Scan 4 Scan 5** | | | | | |
| --- | --- | --- | --- | --- | --- | --- | --- | --- |
| **Medial prefrontal cortex** | F(4,60) = 15.06 | P<0.0001 | 0% | 16±3%******* | 21%±3%******* | 13%±3%****** | | 10%±3%***** |
| **Anterior cingulate cortex** | F(4,60) = 5.45 | P=0.0008 | 0% | 8%±2%****** | 11%±2%******* | | 8%±2%***** | 5%±3% |

Changes in normalized resting cerebral blood flow in mPFC and ACC during (scan 2, 3, and 4) and after (scan 5) infusion of S-ketamine compared to pre-infusion (scan 1) (n=16). ^1^Statistical significance of post hoc tests defined as: * p<0.0125, **p<0.0025, and ***p<0.00025 (p/ 4 to correct for multiple comparisons). ^2^Multivariate test (Pilai’s trace) due to violation of the assumption of sphericity. SEM: Standart error of the mean. rCBF: Resting cerebral blood flow.
