# Supplementary material for: Glutamate Levels and Resting Cerebral Blood Flow in Anterior Cingulate Cortex Are Associated at Rest and Immediately Following Infusion of S-Ketamine in Healthy Volunteers
Source: Front Psychiatry. 2018 Feb 6;9:22. doi: 10.3389/fpsyt.2018.00022 (PMC5808203; doi:10.3389/fpsyt.2018.00022)
Supplement: Supplementary file 7 [file Table_6.doc]

**Table S6: Normalized resting cerebral blood flow in subcortical regions of interest**

| **Region of interest** | | **Statistics,**  **main effect** | | **p,**  **main effect** | | **% rCBF increase ± SEM and post hoc tests^1^**  **Scan 1 Scan 2 Scan 3 Scan 4 Scan 5** | | | | |
| --- | --- | --- | --- | --- | --- | --- | --- | --- | --- | --- |
| **Thalamus, left**  **Thalamus, right** | F(4, 12) = 4.08^2^  F(4, 60)= 0.28 | | P= 0.026  P= 0.89 | | 0%  0% | | -4±2%  -2±2% | 1±3%  0±2% | 2±3%  1±3% | 2±4%  1±4% |
| **Caudate, left** | F(4, 12)= 0.85**^2^** | | P= 0.52 | | 0% | | 1±2% | 2±2% | 2±3% | 6±3% |
| **Caudate, right** | F(4, 60)= 0.37 | | P= 0.83 | | 0% | | 1±2% | 4±3% | 2±3% | 4±3% |
| **Accumbens, left**  **Accumbens, right** | F(4, 12) = 2.00^2^  F(4, 12)=0.56**^2^** | | P= 0.16  P= 0. 40 | | 0%  0% | | 5±2%  5±3% | 4±2%  7±5% | 7±4%  9±5% | 8±4%  9±4% |
| **Putamen, left** | F(4,12)= 0.80**^2^** | | P= 0.55 | | 0% | | 0±2% | -2±2% | 0±2% | -2±3% |
| **Putamen, right** | F(4,12)= 0.15**^2^** | | P= 0.97 | | 0% | | -1±2% | 0±3% | 1±3% | 2±3% |

Changes in normalized resting cerebral blood flow in subcortical regions of interest during (scan 2, 3, and 4) and after (scan 5) infusion of S-ketamine compared to pre-infusion (scan 1) (n=16). ^1^Statistical significance of post hoc tests defined as: * p<0.0125, **p<0.0025, and ***p<0.00025 (p/ 4 to correct for multiple comparisons)**.** ^2^Multivariate test (Pilai’s trace) due to violation of the assumption of sphericity. SEM: Standart error of the mean. rCBF: Resting cerebral blood flow.
